# Few-emitter lasing in single ultra-small nanocavities

**DOI:** 10.1515/nanoph-2023-0706

**Published:** 2024-01-19

**Authors:** Oluwafemi S. Ojambati, Kristín B. Arnardóttir, Brendon W. Lovett, Jonathan Keeling, Jeremy J. Baumberg

**Affiliations:** NanoPhotonics Centre, Cavendish Laboratory, Department of Physics, University of Cambridge, JJ Thompson Avenue, Cambridge, CB3 0HE, UK; SUPA, School of Physics and Astronomy, University of St Andrews, St Andrews KY16 9SS, UK

**Keywords:** nanocavity, plasmonic nanocavity, nanolaser, emitter, nonlinear light emission

## Abstract

Lasers are ubiquitous for information storage, processing, communications, sensing, biological research and medical applications. To decrease their energy and materials usage, a key quest is to miniaturise lasers down to nanocavities. Obtaining the smallest mode volumes demands plasmonic nanocavities, but for these, gain comes from only a single or few emitters. Until now, lasing in such devices was unobtainable due to low gain and high cavity losses. Here, we demonstrate a form of ‘few emitter lasing’ in a plasmonic nanocavity approaching the single-molecule emitter regime. The few-emitter lasing transition significantly broadens, and depends on the number of molecules and their individual locations. We show this non-standard few-emitter lasing can be understood by developing a theoretical approach extending previous weak-coupling theories. Our work paves the way for developing nanolaser applications as well as fundamental studies at the limit of few emitters.

Lasing occurs when stimulated emission into a cavity mode exceeds loss, leading to amplification. Typically, this causes a sharp change of slope in the emission versus input power. Such a sharp transition is analogous to a thermodynamic phase transition in systems in thermal equilibrium [1]. As with such equilibrium phase transitions, a sharp transition only occurs in a thermodynamic limit; away from this limit, the transition is replaced by a smooth crossover. For equilibrium phase transitions, the parameter controlling this is the system size. For a standard lasing transition, with many emitters, there is still a ‘system-size’ parameter *β* (discussed further below), which determines how sharp the transition is [2]. However, further questions arise when one considers systems with few emitters, small cavities and stronger light–matter coupling. Such questions are particularly important for nanocavities, which confine light within sub-wavelength volumes [3], such as metasurfaces or plasmonic nanocavities that exploit collective electron oscillations in metallic nanostructures to achieve extreme confinement (*V* < 100 nm^3^) [4], [5]. These enable coupling single emitters to light [6], [7], [8]. Lasing in such plasmonic nanocavities presents new opportunities for miniaturisation and integration but also raises new questions about the nature and conditions required for such ‘few-emitter lasing’ in a regime, which combines a small number of strongly coupled emitters with lower quality resonators, *Q* ∼ 10. Our aim here is to understand this regime.

In general, the sharpness of the lasing transition reflects how lasing enhances the conversion efficiency of input power into output light. Above threshold, the efficiency is high, as stimulated emission directs almost all radiation into the cavity mode. A sharp transition requires low efficiency below threshold. This is captured by the parameter *β*, the ratio of input–output slopes below and above threshold. Small *β* indicates a sharp transition. When the light–matter coupling *g* is weak, $\beta =g^{2}/\left(g^{2}+\Gamma_{↓}\Gamma_{T}\right)$ , where Γ_*T*_ is the total emitter linewidth, and Γ_↓_ the decay rate into non-cavity modes [2]. When below threshold, *β* is equal to the fraction of incoherent emission into the cavity. For a standard laser (with weak light–matter coupling), the value of *β* fully determines the shape of the input–output curve. It is notable that *β* does not depend on the number of emitters, whereas the parameter determining the sharpness of thermodynamic phase transitions is typically the system size. As such, even for a large number of emitters, the lasing transition can still become a crossover if *β* is large. As we discuss below, allowing for strong light–matter coupling changes this behaviour considerably.

Some extreme limits of lasing have previously been explored. The single-emitter limit [9] has been studied with atoms [10], superconducting circuits [11], and quantum dots [6], [12]. In this limit, the transition broadens as *β* necessarily becomes large. This is because lasing with *N* emitters requires *NC* > 1, where the cooperativity *C* = 4*g* ^2^/(*κ*Γ_*T*_) depends also on the cavity loss rate *κ*. When *N* = 1, one needs *C* > 1, and so a sharp lasing threshold (*β* ≪ 1) is only possible if *κ* ≪ Γ_↓_, which is not typically the case.

In this paper, we explore few-emitter lasing of organic molecules coupled to a plasmonic nanocavity. Despite low *Q*, emitters coupled to plasmonic modes can achieve lasing [13]. However, the smallest lasers must lase with only a few emitters, a goal so far thwarted, but attainable by using ultrasmall volume plasmonic nanocavities. These can be realised using bottom-up self-assembly, which we achieve via the nanoparticle-on-mirror (NPoM) geometry (Figure 1a) of a Au nanoparticle on a thin dielectric spacer above a Au film [5]. We trap light inside 1 nm-high ∼ 20 nm-wide gaps, which enhance incident optical fields by > 250 while retaining ∼50% radiative efficiency. Enhancing both the pumping and the light–matter coupling now enables a form of few-emitter lasing, which we characterise in the following. To understand and describe such experiments, a comparison to theory is key. This is because, as noted above, such few-emitter lasing systems differ from standard textbook lasers. We, therefore, extend previous theoretical treatments [2] to consider the combination of stronger coupling, few emitters, bad cavities, pumping-induced noise, and disorder.

**Figure 1:**
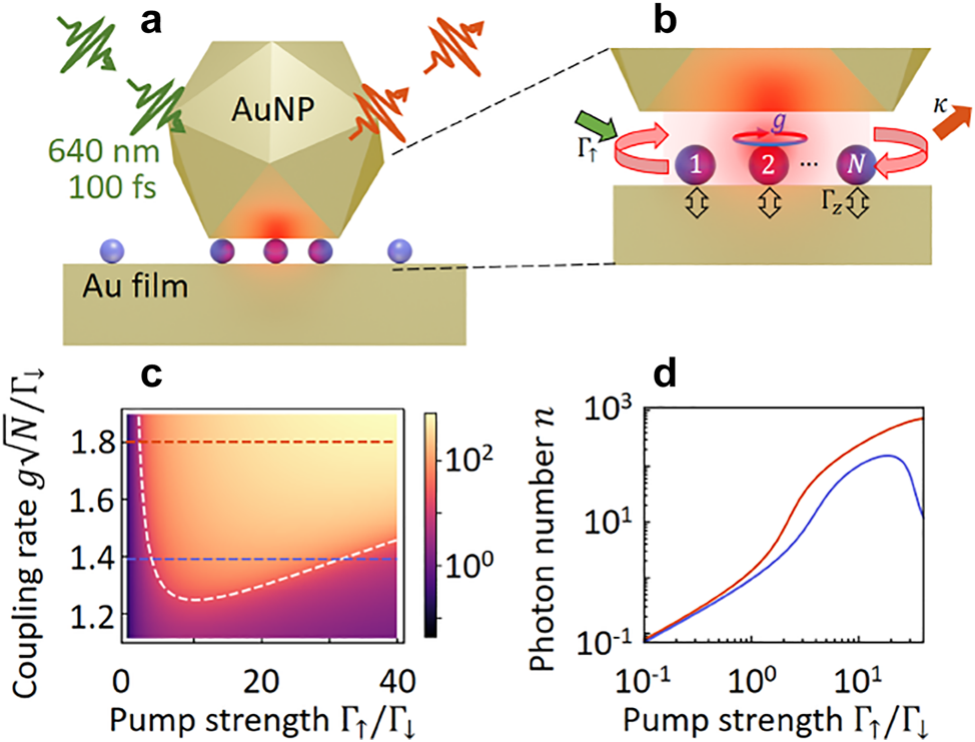
Plasmonic nanocavity with emitters. (a) Nanoparticle-on-mirror (NPoM) cavity formed by Au nanoparticle trapping few emitters in 0.9 nm gap above Au film. (b) Nanogap region with *N* emitters of dephasing Γ_*z*_ excited by pump strength Γ_↑_, emitting into cavity with loss rate *κ*. (c) Re-entrant lasing threshold, shown as colourmap of photon number (yellow = high, purple = low) versus normalised coupling and pump strength, using *κ* = 0.1Γ_↓_, *N* = 10 and Γ_*z*_ = 10Γ_↓_. Dashed white line shows threshold defined by Eq. (3). (d) Cross sections, as indicated by horizontal dashed lines in (c). At large coupling, the traditional input–output curve is seen (red line), while at smaller coupling, lasing is suppressed at higher pump power (blue line).

We model the light–matter interaction in this NPoM nanocavity using a standard master equation describing many two-level molecules coupled to light with strength *g*_*i*_. The molecules undergo incoherent pumping, loss, and pure dephasing at rates Γ_↑_, Γ_↓_, Γ_*z*_ (see Methods, Figure 1b). We then make a second-order cumulant expansion [14], giving coupled equations for photon number *n*, molecule–photon coherence, inter-molecular coherence and molecular inversion. This approach is ideal for understanding how system size *N* controls the sharpness of the transition, since it captures finite size effects treating *N* as a parameter. It also includes the effects of spontaneous emission and recovers the semiclassical theory of lasing [2], [15] in appropriate limits. In contrast to weakly coupled models of incoherent emission and absorption [2], our approach captures the back-action of the coherent light on the dynamics of the emitters.

Considering first the homogeneous case where *g*_*i*_ = *g*, we solve for steady-state *n* with constant pump Γ_↑_ giving

(1) $$n=\frac{n_{0}}{2}\left[\left(\frac{\Gamma_{↑}}{\Gamma_{c}}-1\right)+\sqrt{{\left(\frac{\Gamma_{↑}}{\Gamma_{c}}-1\right)}^{2}+4\beta \left(\Gamma_{↑}\right)\frac{\Gamma_{↑}}{\Gamma_{c}}}\right]$$

with *n*_0_ defined in the Methods, and

(2) $$\beta \left(\Gamma_{↑}\right)=\frac{2\kappa \Gamma_{T}}{N\left(\kappa +\Gamma_{T}\right)}\frac{{\left(NC\right)}^{2}}{{\left(NC-1\right)}^{2}\Gamma_{c}},$$

(3) $$\Gamma_{c}=\frac{NC+1}{NC-1}\Gamma_{↓}+\frac{NC}{NC-1}\frac{\kappa \Gamma_{T}}{N\left(\kappa +\Gamma_{T}\right)},$$

where the total linewidth is Γ_*T*_ = Γ_↑_ + Γ_↓_ + 4Γ_*z*_. While homogeneous *g*_*i*_ = *g* gives a simple result here, as discussed below, the real experiment is inevitably affected by disorder. We show the effects of this in the discussion below, and the Supplementary Information (SI) extensively discusses the theoretical model with disorder.

While Eq. (1) appears identical to that found in [2], a subtle and crucial difference exists: the terms Γ_*c*_ and *β* both implicitly depend on Γ_↑_, because the cooperativity *C* depends on Γ_*T*_. This Γ_↑_ dependence can often be ignored, when Γ_*T*_ ≃ 4Γ_*z*_. However, in the regime where the collective cooperativity is low (as here), it is important to keep this dependence. Physically, strong pumping introduces phase noise, which ultimately kills lasing by suppressing the cooperativity. For typical lasers, this effect only occurs at very high powers; however, for plasmonic nanocavities with high losses, a switch-on and switch-off transition are seen, and these can merge where *NC* ≃ 1 [16]. This is seen in Figure 1(c and d), which show how the photon number depends on the normalised pump power and coupling strength.

A second crucial difference from [2] is the form of *β* in Eq. (2). In particular, this now depends on *N*, with *β* ∼ 1/*N* at large *N*. This encodes the anticipated effect that larger systems show smaller finite-size effects.

Experimentally, here NPoMs with ∼0.9 nm gap are defined by a monolayer of cucurbit[7]uril molecules that can each encapsulate a single molecule of methylene blue [7] (for sample preparation see Methods). We vary the average number of emitters $\bar{N}$ in the nanocavity by changing the ratio of emitting methylene blue to non-emitting cucurbit[7]uril molecules. Because the location of the emitting molecules is different in each NPoM, their overlap with the nanocavity mode and hence coupling *g*_*i*_ varies. This disorder in couplings means that the behaviour in each realisation will differ. We, therefore, show results from an ensemble of many measurements with the same average number of emitters. Using NPoM nanoparticle diameters of 60 nm tunes the dominant NPoM cavity mode to the gain peak (Figure 2a), optimising the interaction of the emitted light with the nanocavity.

**Figure 2:**
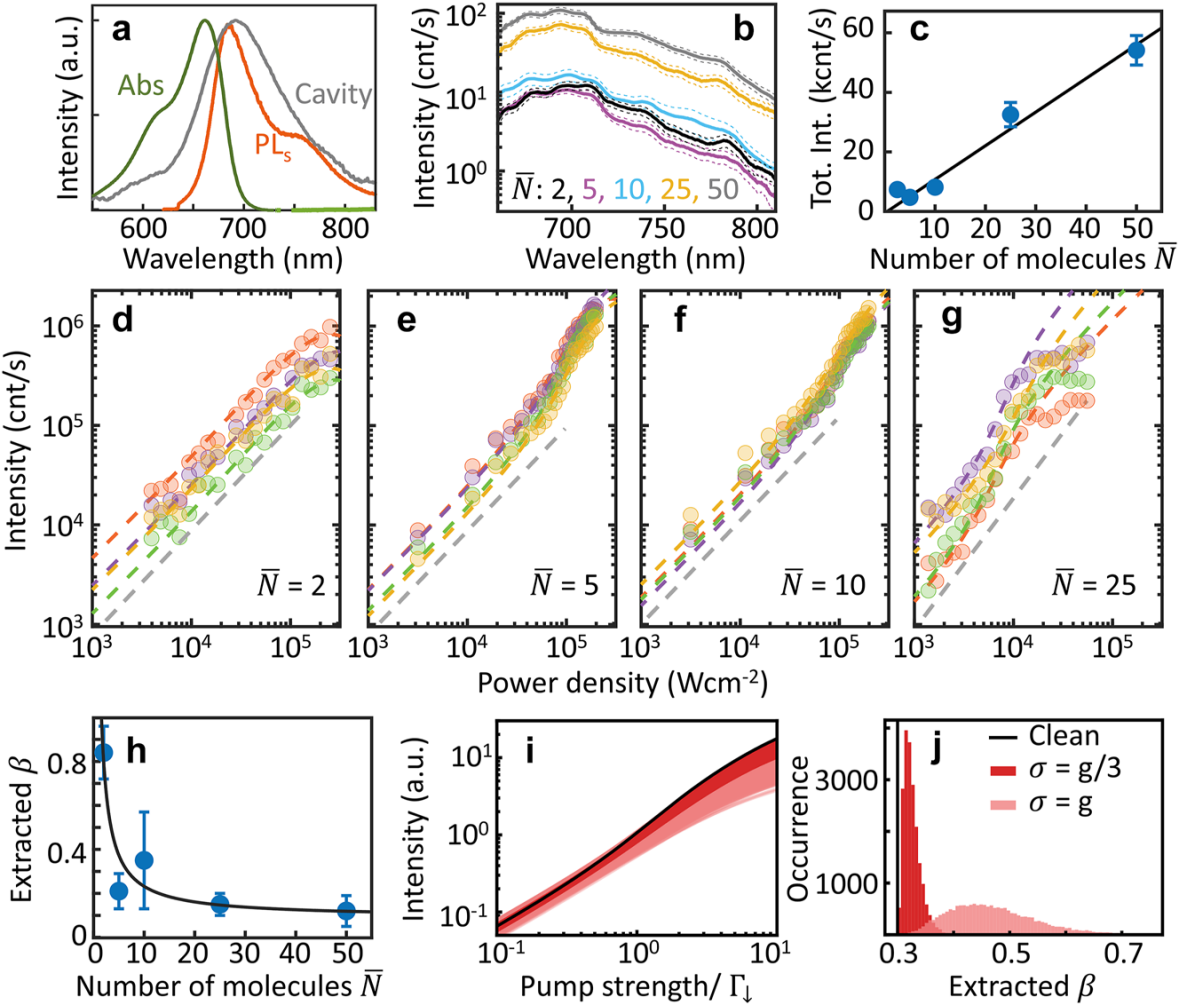
Emission dependence on excitation power and number of emitters. (a) Absorption (green) and emission (orange) spectra of methylene blue in solution, and typical NPoM darkfield scattering spectrum (grey). (b) Dashed lines: example emission spectra for different expected number of emitters $\bar{N}$ in NPoM. Solid curves are averaged over > 50 NPoMs. (c) Spectral integrated intensity versus $\bar{N}$ (blue circles) and linear fit (solid curve). Error bar is the standard error. (d–g) Spectral integrated intensity versus power density for different $\bar{N}$ , colours correspond to different NPoMs. Dashed coloured lines are theoretical fits (see Methods) and the dashed grey lines are guide-to-the eye linear trends, which clearly deviate from the $\bar{N}>2$ data. (h) Extracted slope ratio $\beta \;\text{versus}\;\bar{N}$ with predicted theoretical trend (solid curve), bars give range. (i and j) Theoretical input–output curves, and extracted distribution of *β*, showing effect of disorder in couplings *g*_*i*_. Black line shows the homogeneous limit, *g*_*i*_ = *g* = 1.5Γ_↓_ for *N* = 10 emitters, with Γ_*z*_ = Γ_↓_, *κ* = 1.74Γ_↓_. The red and pink curves (and corresponding distributions of *β*) arise by sampling *g*_*i*_ from a Gaussian distribution with root mean square *g* = 1.5Γ_↓_ and standard deviation *g*/3, (*g*) respectively. The thick black line corresponds to the homogeneous case, where no disorder is present.

To create enough gain, ultrafast pulses (100 fs, 640 nm) are used to irradiate individual NPoM cavities whose emission is recorded by a low-noise spectrometer (for experimental setup see Methods). At an average power of *P*_av_ = 4 *μ*W (power density = 3.2 kW cm^−2^), the emission spectra for different numbers of molecules is similar and the total integrated intensity varies linearly with $\bar{N}$ (Figure 2b and c).

Power-dependent measurements on multiple NPoM constructs are performed with different $\bar{N}$ (Figure 2d–g). For NPoM cavities with lower $\bar{N}$ , smooth transitions in the total emitted intensity are seen, becoming systematically sharper for higher $\bar{N}$ . In particular for $\bar{N}∼2$ , no observable transition is seen and the total emission is linear before saturating at the highest powers. This is consistent with thresholdless few-emitter lasing, approaching the single-emitter regime. For NPoMs with large $\bar{N}$ , well-pronounced thresholds are seen with super-linear emission, which then saturate to linear scaling as in conventional lasers. We convert the emitted power in count/s to W and find that the NPoM laser emits order-of-magnitude pW average powers, after accounting for the optical losses in the beam path. We fit Eq. (1) to the experimental data (for fitting procedure see Methods); our theory matches well across most of the range of $\bar{N}$ , indicating few-emitter lasing occurs in plasmonic nanocavities. Furthermore, the extracted *β*-factor varies inversely with $\bar{N}$ (Figure 2h), as expected from our theoretical model (Eq. (2)). For $\bar{N}=25$ , experiments deviate from the theoretical curves at large powers. This could result from intermolecular quenching, where two excitations in close proximity can lead to enhanced loss. Such a process is expected to become stronger when emitters are closer, as occurs for larger $\bar{N}$ . See Supplementary Information for further discussion.

The analytic form of Eq. (1) ignores the effects of random placement of molecules, which gives variable light–matter coupling. This variation becomes important when the number of molecules is small, as it leads to significant variation between different NPoMs with the same number of molecules. With random positions, one finds that the shape of the input–output curve (and thus the parameter *β*) depends on the distribution of couplings, as shown in Figure 2(i and j).

Because the threshold is less defined at small *N*, it becomes important to find further experimental evidence for few-emitter lasing. To this end, we examine the spectral evolution and coherence across the transition. As the average power *P*_av_ increases from 4 μW to 250 μW (Figure 3a), the emission (which is 10 nm red-shifted from the solution PL due to the cavity environment) significantly broadens, with increasing weight at shorter (bluer) wavelengths. This behaviour appears consistently in most NPoMs at all $\bar{N}$ , with emission even extending to the blue side of the pump laser wavelength (see SI). Such blue-shifted emission indicates emission occurring from vibrationally excited states. This is in contrast to Kasha’s rule [18], which states emission occurs from the vibrational ground state. There are several reasons this rule might break down at increasing pump power. One possible cause would be an increased emission rate. Both Purcell enhancement and stimulated emission (the latter of which increases with pumping) speed up the emission. If emission becomes faster than the vibrational lifetime (∼1 ps) [8], molecules do not relax before emission, allowing direct emission from higher vibronic states as previously proposed for strongly coupled systems [19]. Another explanation is heating of the molecules, e.g. by the creation of hot electrons at high pump intensities. Since (as discussed in the Supplementary Information) emission can be seen at energies *higher* than the excitation wavelength, lack of relaxation cannot be the full explanation; some amount of heating, which can cause emission at such higher energies, at high powers seems also likely.

**Figure 3:**
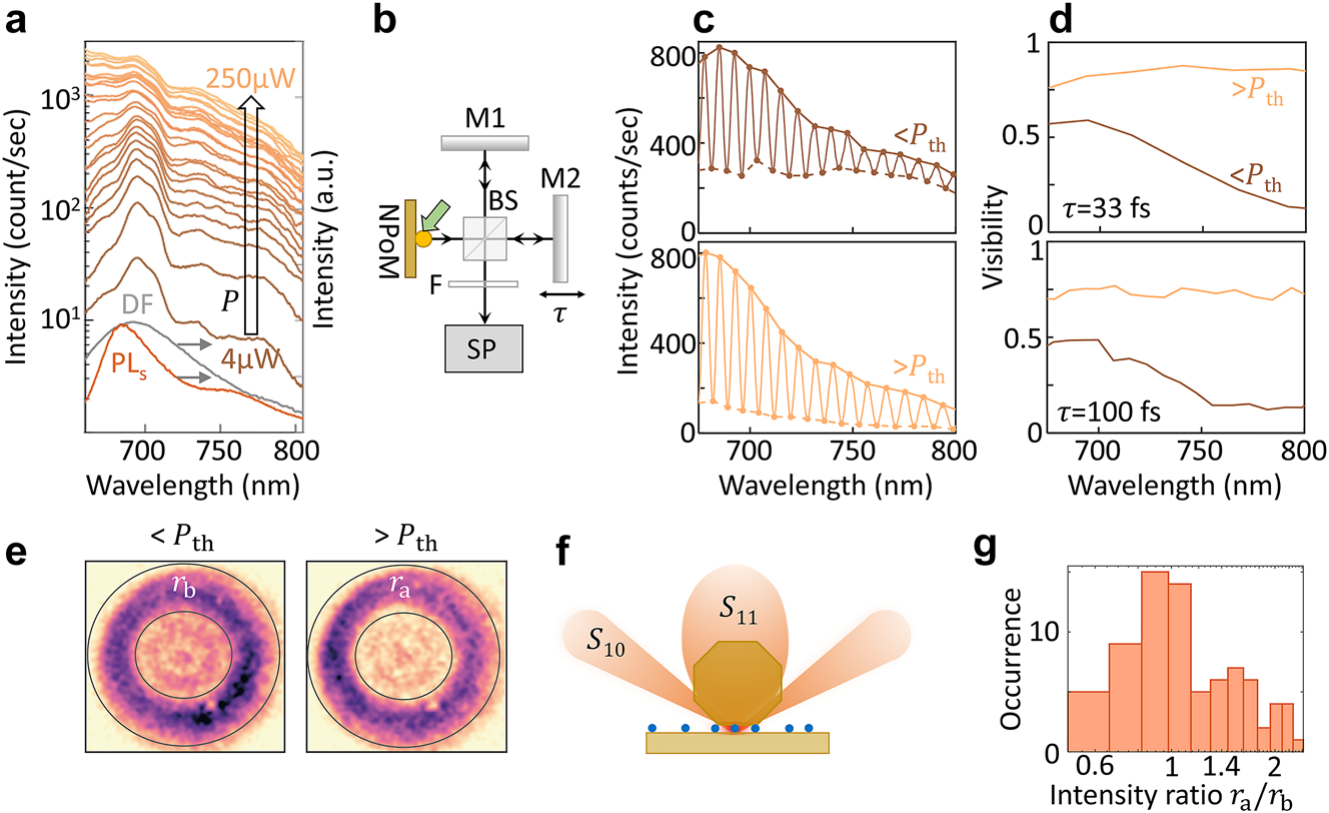
Emission spectra and coherence of nanolaser. (a) Emission spectra for increasing excitation powers *P*, with dark-field (DF) and solution PL. (b) Schematic of Michelson interferometer to measure spectral coherence. Emission from NPoM is split in two by 50:50 beamsplitter (BS), reflected from mirrors (M1, M2) with delay in one arm and recombined, filtered (F) and sent to spectrometer (SP). (c) Spectral fringes with average powers below (upper panel) and above (lower panel) few-emitter lasing threshold (*P*_th_). (d) Visibility versus wavelength at different delays *τ*. (e) Far-field Fourier space images of emission below and above threshold, divided into annular ring and centre. The integrated intensities in these regions, *I*_ring_ and *I*_centre_ allow us to define an intensity ratio *r* = *I*_ring_/*I*_centre_ both below threshold (*r*_b_) and above threshold (*r*_a_). Numerical aperture 0.9 limits collection angle <64° . (f) NPoM radiative modes *S*_10_ and *S*_11_ that emit at high and low angles, respectively, based on analysis in Ref [17]. (g) Histogram of relative intensities in the high-angle ring *r*_a_/*r*_b_, with a logarithmic scale on the horizontal axis.

Regarding the lack of linewidth narrowing seen in the emission (which is expected for regular lasing), this can be explained by the still small number of photons in the system. Theoretical calculations of few-emitter lasing in our model system show the same lack of narrowing, as discussed in the Supplementary Information (SI Section E).

To track the coherence of the observed emission, we explore spectral interference using a Michelson interferometer (Figure 3b). The delay time *τ* between the two arms of the interferometer is greater than the temporal coherence time (*τ* > *τ*_c_ ∼ ℏ/Γ_*T*_ ∼ 13 fs). Spectral fringes are observed both below and above threshold (Figure 3) with a wavelength period inversely proportional to *τ *(spectral period 7 nm for *τ*=100 fs in Figure 3c). The extracted visibility from the spectral envelope of the fringes decreases with wavelength below *P*_th_ to 0.1 at 800 nm (shown for two different time delays *τ* = {33, 100} fs, in Figure 3d). By contrast, above threshold the visibility increases to 0.8, becoming constant over the entire spectrum.

As discussed in Ref. [20], by combining spectral filtering with coherence measurements, we show in Figure 3c and d how the spatial coherence of the light changes through the threshold. To better understand this increasing visibility, the far-field emission is measured in Fourier space (for optical setup see Methods). The dominant bright plasmonic mode *S*_10_ emits at high angles both below and above threshold (seen as purple-coloured ring in Figure 3e), around 40–50° (Figure 3f). Additional weaker emission at low angles – arising from the *S*_11_ mode [17], [21] – is proportionately larger below threshold. This mode mostly outcouples light from molecules near the edges of the nanoparticle facet (Figure 3f) but only with poor quantum efficiency <5% [21]. We define the emission ratio *r* = *I*_ring_/*I*_centre_ between the intensity from the annular ring *I*_ring_ and from the centre *I*_centre_. This measures the relative emission of these modes. By comparing the emission ratios above and below threshold *r*_a,b_, we see that *r*_a_/*r*_b_ ∼ 1 for some NPoMs but >1 for many others. This indicates preferential occupation of one mode, as is typical in lasers, due to stimulated emission.

To gain further insight into this behaviour, we have included the *S*_11,10_ modes into our theoretical calculations and studied how the relative occupation of the different modes behaves. Figure S12(a) shows the mode purity of the lowest energy mode (*S*_10_) – i.e. how much of the plasmon occupation is in this mode – as a function of pump strength for different realisations of the system. Figure S12(b) then shows the ratio of mode purity at a point above and below the few-emitter lasing threshold. This confirms that indeed linewidth reduction is not expected above the few-emitter lasing threshold in such nanoscale systems.

In summary, we show extreme confinement of optical fields exciting a few molecules in a nanometre gap that reveals few-emitter lasing is possible, and that this can match only extended models. The results shown above demonstrate the existence of a lasing threshold, enhanced coherence and some degree of directionality of emission. Because this few-emitter system differs from a textbook laser, comparison to an appropriate theoretical model is key to check which features of lasing should or should not be expected. In agreement with theory, spectral narrowing is not seen in this few-emitter lasing regime. Another property typically expected of lasers is a change of the photon statistics. These are not resolvable in our experiments due to the ultrafast timescale of emission. We hope that our results will inspire future work exploring the properties from such ultimately miniaturised lasers and clarifying the fundamentals of the few-emitter lasing nonlinear phase transition.

## 1 Methods

### 1.1 Theoretical modelling

In this section, we describe the theoretical approach we use to calculate the input–output curve for lasing with few strongly coupled emitters. We first introduce the model and then discuss the cumulant approach, and how the resulting equations can be solved with and without disorder in the matter–light coupling.

#### 1.1.1 Model

We model the system as a plasmonic mode coupled to a collection of *N* two-level systems:

(4) $$H=\omega a^{†}a+\overset{N}{\underset{i}{\sum }}\left[\frac{\epsilon }{2}\sigma_{i}^{z}+g_{i}\left(a^{†}\sigma_{i}^{-}+a\sigma_{i}^{+}\right)\right],$$

here, *a* ^(†)^ is the annihilation (creation) operator for the plasmonic mode of energy *ω* while $\sigma_{i}^{\alpha }$ are the Pauli matrices representing the two-level system with energy *ϵ* at site *i*. We use a Lindblad master equation to account for the following incoherent processes with their corresponding rates: photon loss (*κ*), incoherent pump (Γ_↑_), non-radiative losses (Γ_↓_) and pure dephasing (Γ_*z*_). We thus have

(5) $$\begin{array}{l} \;\partial_{t}\rho =-i\left[H,\rho \right]+\kappa D\left[a\right]\\ +\underset{i}{\sum }\left(\Gamma_{↓}D\left[\sigma_{i}^{-}\right]+\Gamma_{↑}D\left[\sigma_{i}^{+}\right]+\Gamma_{z}D\left[\sigma_{i}^{z}\right]\right), \end{array}$$

with $D\left[X\right]=X\rho X^{†}+\frac{1}{2}\left(X^{†}X\rho +\rho X^{†}X\right)$ .

The model we consider does not explicitly include the vibronic progression present in organic molecules [14]. The incoherent pumping rate Γ_↑_ we consider should, however, be understood as an effective rate, arising from the combination of coherent pumping exciting a higher vibrational state followed by vibrational relaxation (see Figure 1). This simplified model is chosen since, as discussed below, it allows for closed-form expressions for the lasing threshold. Exploring how more complex treatment of the molecular spectrum affects these results is an important question for future work.

#### 1.1.2 Cumulant equations

Various approaches exist to model a system described by the above equations [22]. In this work, as discussed in the main text, our focus is on understanding how system size *N* enters into determining the sharpness of the transition. For this, it is useful to choose an approach that captures finite size effects, treats *N* as a parameter and captures the semiclassical effects of spontaneous emission. The ideal approach for this is to use second-order cumulants [14], [23], which provide a systematic expansion in 1/*N*.

From the density matrix equation of motion given above, we can write down equations of motion for the second order moments of the system, using cumulant expansion to break higher order moments into a combination of first and second order moments. The non-zero moments are *n* = ⟨*a* ^†^ *a*⟩, $P_{i}=\text{Im}\left\langle a\sigma_{i}^{+}\right\rangle ,S_{i}=\left\langle \sigma_{i}^{z}\right\rangle$ and $D_{ij}=\left\langle \sigma_{i}^{+}\sigma_{j}^{-}\right\rangle$ , where we require the two operators in the last moment to act on different molecules. Assuming the resonant case *ω* = *ϵ*, the equations of motion end up taking the form:

(6) $$\partial_{t}n=-\kappa n-2\underset{i}{\sum }g_{i}P_{i};$$

(7) $$\partial_{t}P_{i}=-\frac{\kappa +\Gamma_{T}}{2}P_{i}-g_{i}\left(nS_{i}+\frac{S_{i}+1}{2}+\underset{j\neq i}{\sum }D_{ij}\right);$$

(8) $$\partial_{t}S_{i}=-\left(\Gamma_{↑}+\Gamma_{↓}\right)\left(S_{i}-S_{0}\right)+4g_{i}P_{i};$$

(9) $$\partial_{t}D_{ij}=-\Gamma_{T}D_{ij}-\left(g_{i}S_{i}P_{j}+g_{j}S_{j}P_{i}\right),$$

where Γ_*T*_ = 4Γ_*z*_ + Γ_↑_ + Γ_↓_ is the total molecular linewidth and $S_{0}=\frac{\Gamma_{↑}-\Gamma_{↓}}{\Gamma_{↑}+\Gamma_{↓}}$ is the inversion set by the pump. Note that in writing these equations, we have allowed molecule-dependent coupling strengths *g*_*i*_.

#### 1.1.3 Homogeneous limit

We will start by considering the case where *g*_*i*_ = *g*, i.e. where all the molecules couple identically to the light mode. If we use Eqs. (6)–(9) to adiabatically eliminate everything but the photon occupation *n*, we end up with the quadratic equation for the steady state:

(10) $$\begin{array}{ll} 0 & =2\kappa \left[\Gamma_{T}+\kappa \left(1-\frac{1}{N}\right)\right]{\left(\frac{n}{N}\right)}^{2}\\ & \;-\left\{\left[\Gamma_{T}+\kappa \left(1-\frac{1}{N}\right)\right]\left(\Gamma_{↑}-\Gamma_{↓}\right)\right.\\ & \;\left.-\frac{\kappa \Gamma_{T}}{N}-\frac{\kappa \Gamma_{T}\left(\Gamma_{T}+\kappa \right)}{4g^{2}N}\left(\Gamma_{↑}+\Gamma_{↓}\right)\right\}\frac{n}{N}-\frac{\Gamma_{T}\Gamma_{↑}}{N}. \end{array}$$

If we make the approximation that $\Gamma_{T}+\kappa \left(1-\frac{1}{N}\right)\approx \Gamma_{T}+\kappa$ , we can write the solution on the form

(1) $$n=\frac{n_{0}}{2}\left[\left(\frac{\Gamma_{↑}}{\Gamma_{c}}-1\right)+\sqrt{{\left(\frac{\Gamma_{↑}}{\Gamma_{c}}-1\right)}^{2}+4\beta \frac{\Gamma_{↑}}{\Gamma_{c}}}\right].$$

Introducing the cooperativity *C* = 4*g* ^2^/*κ*Γ_*T*_, we can write the quantities appearing in Eq. (1) as:

(11) $$\Gamma_{c}=\frac{NC+1}{NC-1}\Gamma_{↓}+\frac{NC}{NC-1}\frac{\kappa \Gamma_{T}}{N\left(\kappa +\Gamma_{T}\right)};$$

(12) $$n_{0}=\frac{N\Gamma_{c}}{2\kappa }\frac{NC-1}{NC};$$

(13) $$\beta =\frac{2\kappa \Gamma_{T}}{N\left(\kappa +\Gamma_{T}\right)}\frac{{\left(NC\right)}^{2}}{{\left(NC-1\right)}^{2}\Gamma_{c}}.$$

As noted in the main text, the dependence of Γ_*T*_ on Γ_↑_ means the input–output curve takes a more complex form than discussed by [2].

#### 1.1.4 Effects of inhomogeneity

In reality, the electric field strength at the location of the molecules (and thus their coupling to light) will not be identical. This becomes especially important when the molecules are few and the mode volume is very small, as is the case in this paper. We next discuss the effects of such disorder. To do this, we reintroduce an *i*-dependence of the coupling strength *g*_*i*_ in the cumulant equations. In this case, to find the steady state, it is convenient to first solve for *P*_*i*_. This must satisfy the quadratic equation $0=A_{i}P_{i}^{2}+B_{i}P_{i}+C_{i}$ with coefficients that depend on the other *P*_*j*_ via the moments $\Pi^{\left(n\right)}=\sum_{i}g_{i}^{n}P_{i}$ . The coefficients take the form:

$$\begin{array}{ll} A_{i} & =16g_{i}^{3}\\ B_{i} & =\Gamma_{T}\left(\Gamma_{T}+\kappa \right)\left(\Gamma_{↑}+\Gamma_{↓}\right)\\ & \;+2g_{i}\left[2g_{i}\Gamma_{T}\left(1-\frac{4}{\kappa }\Pi^{\left(1\right)}\right)-\left(\Gamma_{↑}-\Gamma_{↓}\right)\left(\tilde{g}-2g_{i}\right)\right.\\ & \left.\;-4\left(\Pi^{\left(2\right)}+g_{i}^{2}\Pi^{\left(0\right)}\right)\right]\\ C_{i} & =2g_{i}\left[\Gamma_{T}\Gamma_{↑}-\frac{2\Gamma_{T}\left(\Gamma_{↑}-\Gamma_{↓}\right)}{\kappa }\Pi^{\left(1\right)}-\left(\Gamma_{↑}-\Gamma_{↓}\right)g_{i}\Pi^{\left(0\right)}\right], \end{array}$$

For a given set {*g*_*i*_}, we can solve these equations iteratively. We first guess an initial {*P*_*i*_} and then evaluate the moments Π^(*n*)^, *n* = 0, 1, 2 to find the coefficients *A*_*i*_, *B*_*i*_ and *C*_*i*_. We then solve the quadratic equation to give a new set {*P*_*i*_}. This process can then be iterated to convergence. We find it necessary to use successive over-relaxation to improve the stability of this.

In Figure 2(i and j), we show the result of this, where we choose a set of *g*_*i*_ drawn from a normal distribution of a given mean and variance. In plotting these, we in fact adjust the mean of this distribution so as to keep the root mean squared *g* constant, since the behaviour of strongly coupled systems typically depends on $\sum_{i}g_{i}^{2}$ . Figure 2(i) shows a set of input output curves; one sees that with disorder, each realisation leads to a slightly different curve. Figure 2(j) is a histogram showing the frequency of slope ratios *β* extracted from these curves, which is, therefore, the resulting probability distribution of this parameter.

### 1.2 Experimental setup

The details of the setup can be found in Ref. [8] and we give a brief description here. For the power-dependent measurements, we used a variable neutral density filter to control the intensity of 100 fs pulses at wavelength 640 nm generated from a tunable optical parametric oscillator (SpectraPhysics, Inspire), pumped at 820 nm with a repetition rate of 80 MHz (SpectraPhysics, Maitai). A brightfield/darkfield microscope objective (Olympus, 100×, numerical aperture = 0.9) focusses the attenuated pulses on a single NPoM nanocavity to excite the emitters. The emitted light was collected in the reflection direction, filtered and measured using a grating spectrometer (Andor, Shamrock 303i) and charged-coupled detector (CCD, Andor iDus).

The farfield Fourier space imaging was performed by demagnifying the collected emission by the objective and then focussing it in the focal plane of a lens before the entrance of a monochromator slit. Darkfield spectroscopy was performed on a home-built microscope that illuminates the sample with white light from a halogen lamp (Philips, 100 W) at angle of >64° through the darkfield microscope objective. The scattered light was collected through the same microscope objective and the scattered spectrum was measured using a fibre-coupled grating spectrometer (OceanOptics).

### 1.3 Sample preparation

The sample substrate is a Au mirror on a Si wafer that was fabricated using a template-stripping process. Thermal evaporation was used to coat a Si wafer with a 100 nm thick Au film at an average evaporation rate of 0.5 Å/s. Small pieces of another Si wafer (area 4 × 4 mm^2^) were glued to the evaporated Au using epoxy (Epo-Tek 377), left overnight at 150 °C and then slowly cooled down to room temperature. On applying a slight force, the silicon pieces easily peeled off and were covered by an atomically flat Au film. A 1 mM solution of methylene blue (Sigma Aldrich) and a 1 mM solution of cucurbit[7]uril (CB) (Sigma Aldrich) were mixed together, thus allowing the encapsulation of the methylene blue guest molecules inside the CB cavities. A freshly stripped substrate with Au film was submerged in the solution overnight, thoroughly rinsed with de-ionised water and then blown dry with nitrogen, leaving behind a self-assembled monolayer (SAM) of CB encapsulating a single molecule of methylene blue  [7]. Au nanoparticles (BBI Solutions, diameter 60 nm) were drop cast onto the SAM on the Au substrate for 10 s. The excess Au nanoparticle solution was rinsed with water and blown dry using nitrogen. This leaves sparsely spaced Au nanoparticles deposited on the CB layer on the Au film, forming the NPoM nanocavity. To control the number of emitters in the nanogap, we diluted the 1 mM methylene blue solution by factors of 2–20 and kept the CB concentration fixed at 1 mM. Using the surface packing density of CB of 0.24 mol nm^2^ [21] and a facet size of 15 nm, we estimated the average number of molecules in the nanogap to vary between 2 and 50 for different dilution ratios.

### 1.4 Fitting of theoretical curves to experimental data

In Figure 2(d–g), we present results of fitting the theoretical expression Eq. (1) to the experimental input–output curves. Here, we describe the approach used to this fitting.

To perform such a fit, one must relate the theoretical input Γ_↑_ and output *n* to the experimental power in and power out. This requires a description of how light couples into and out of the coupling. The in-coupling is taken to be proportional to the coupling strength squared, Γ_↑_ ∝ *g* ^2^ × Power density. This is because the external pump couples to the emitters through the same dipole moment and mode profile as controls the light–matter coupling. As we assume this in-coupling is an incoherent absorption process, Fermi’s golden rule implies a rate proportional to *g* ^2^. Including this effect was crucial in producing an accurate fit; without this factor, our fitting led to spurious correlations between fitting parameters. Out-coupling is simpler, as this involves how the light in the cavity couples to the far-field. This was assumed to be independent of other parameters and considered as an intensity scaling factor.

With the above assumptions, we then performed a standard least squares fit to find the remaining model parameters. Some of these parameters are taken as global parameters – common to all experiments; these were Γ_*z*_, Γ_↓_, *κ* and were first optimised (see SI). The remaining fitting parameters for each NPoM are *g*, an intensity scaling factor and *N*, which only varies by 20 %, due to variations in the number of emitters. The results of the fittings show that there is no correlation between the fitting parameters and that the out-coupling efficiency is linearly proportional to the output intensity, as expected (for more details, see SI).

## Supplementary Material

Supplementary Material Details
