# Supplementary material for: Few-emitter lasing in single ultra-small nanocavities
Source: Nanophotonics. 2024 Jan 19;13(14):2679–86. doi: 10.1515/nanoph-2023-0706 (PMC11147496; doi:10.1515/nanoph-2023-0706)
Supplement: Supplementary file 1 — Supplementary Material Details [file j_nanoph-2023-0706_suppl_001.pdf]

PICOFORCE (Grant Agreement No. 883703), THOR (Grant Agreement No. 829067) and POSEIDON (Grant Agreement No. 861950). O.S.O acknowledges the support of a Rubicon fellowship from the Netherlands Organisation for Scientific Research. We thank Rohit Chikkarddy and Rakesh Arul for valuable discussions and Jack Griffiths for making the cartoon in Fig. 1.

SUPPLEMENTARY INFORMATION

A. Sample characterization using darkfield scattering

We prepared several nanoparticle-on-mirror (NPoM) samples with different numbers of methylene blue emitters, that are each encapsulated inside cucurbit[7]uril, defining the nanogap to be 0.9 nm (for sample preparation, see Methods). We characterised the samples using darkfield scattering, which illuminates the NPoM nanostructure at high angles $> 60°$ and collected only low-angle scattering from the nanostructure. The high angle illumination helps to reduce specular reflections from the Au film, thus the collected intensity is largely from the nanoparticle. We measured many NPoM samples for different number of emitters (N) and plot the histogram of the scattering spectra and representative spectra from each histogram bin (Fig. S1, a-e). The scattering spectra show a dominant plasmonic mode that is nearly constant over different N (Fig. S1 f) with a mean value at 686 nm. Using a plasmonic circuit model, this scattering peak corresponds to a 1 nm nanogap, with a 60 nm Au nanoparticle and assuming a refractive index of 1.6 [24]. This scattering peak is consistent with previous measurements from similar samples [7, 21].

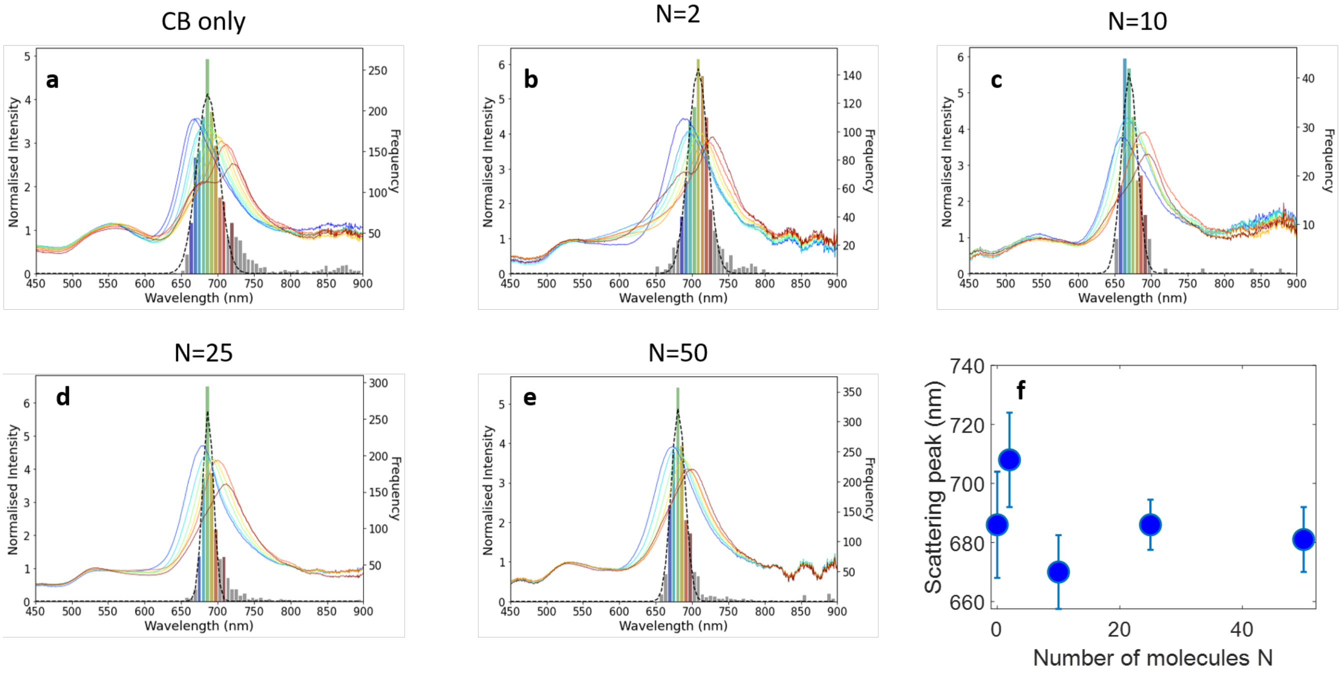

FIG. S1. (a-e) Scattering spectrum of nanoparticle-on-mirror structures. Panel (a) shows the case with only cucurbit[7]uril (CB), providing the gap, while panels (b-e) show varying numbers of emitter molecules. (f) Scattering peak *vs* number of molecules N.

B. Power dependent measurements

The emission spectra were measured from many NPoMs samples. The measurements were controlled and automated using a custom Python software that used the darkfield scattering image to locate a single NPoM, measure the darkfield spectrum, rotate a variable neutral density filter to control the laser power, and measure the emission spectrum (for optical setup, see Methods). This procedure was repeated for > 50 NPoMs and some examples of the spectrally-integrated intensity are shown in Fig. S2.

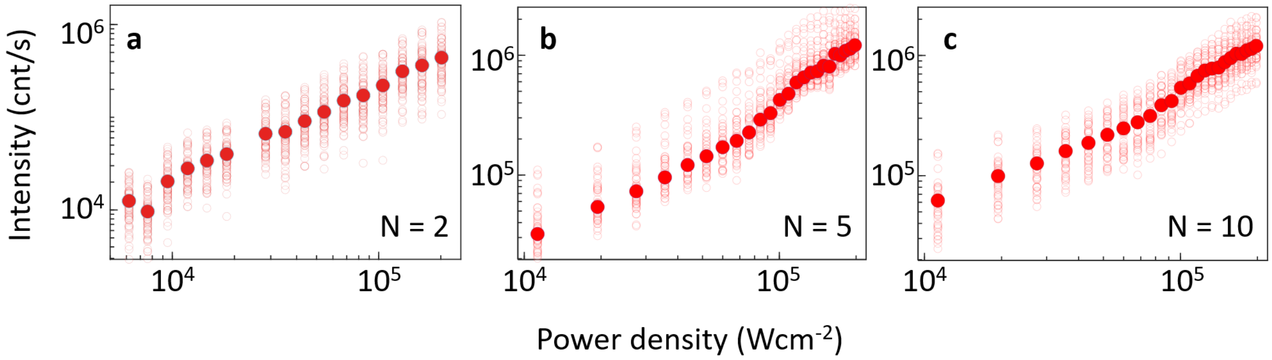

FIG. S2. (a-c) Input-output curves of several NPoM samples (> 50, open red circles) each for different number of molecules N. The filled red circles are the averages over all the NPoM samples

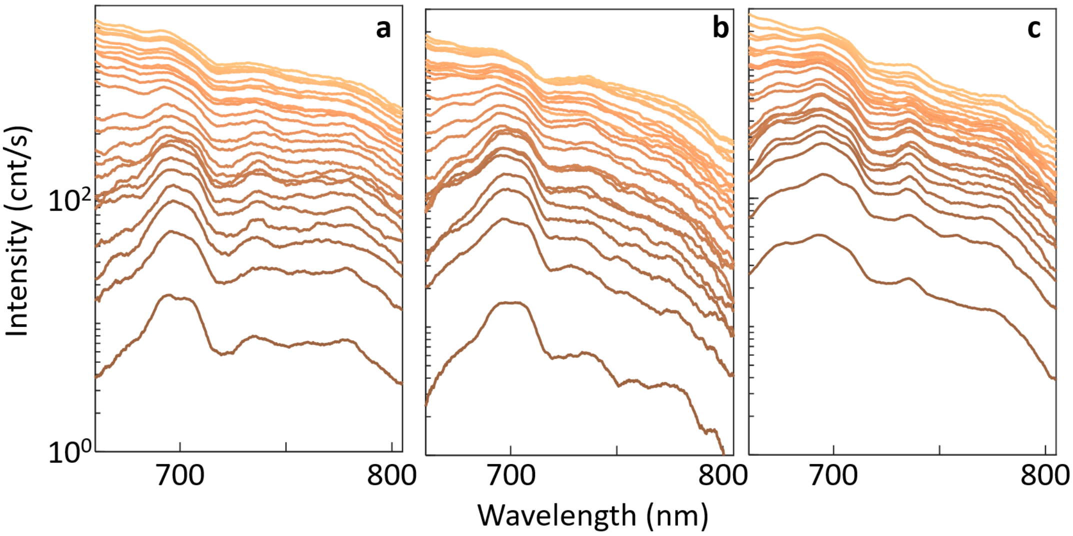

FIG. S3. (a-c) Examples of emission spectra for increasing pump power from three different NPoMs with $N = 5$.

As the laser power increases from 4 μm to 250 μm, the emission spectra gradually change shape, with the bluer region of the spectra increasing and dominating at the highest powers (Fig. S3). This effect consistently occurs for several NPoMs with different number of emitters. The increase in the bluer emission implies that emission occurs directly from excited vibronic states. This could result from several mechanisms. It could be heating due to e.g. hot electrons created at high pump intensities. It could also be a breakdown of Kasha's rule, due to enhanced emission rate, such that emission is faster than vibrational relaxation. Emission rates can be enhanced both by the high Purcell factors in the nanogap, by a factor of up to 10^5 [7, 25], and theoretical work on strongly coupled organic molecules has shown a breakdown of Kasha's law can result [19]. In addition to Purcell enhancement, stimulated emission leads to a further (pump-dependent) increase in emission rate. An explanation based on enhanced emission rate would suggest emission from states that match the excitation energy. As shown in figures further below, we also see emission at higher energies at the highest pump strengths—i.e. anti-Stokes emission. This implies heating must play a role, to allow for emission at higher energy than excitation. A comparison of the emission and surface-enhanced resonant Raman scattering from the NPoM does not show any corresponding peaks between the SERS and emission (Fig S4), implying that the ground state vibrational levels are not involved in the process. Even though the two processes - fluorescence and resonant Raman - are two competing effects, fluorescence emission is more efficient due to the lifetime (50 fs), which is about 30 times faster than the vibrational lifetime of 1.5 ps [26]. It would therefore be interesting in future to investigate the impact of the various vibronic states on the light-matter interactions in this lasing regime [27].

Futhermore, we analyzed the emission over different spectral regions: 660 nm – 715 nm and 715 nm – 850 nm. Both spectral regions have similar trends (Fig. S5) and the nonlinear emission persists over the entire spectrum. In addition, we measured the emission spectrum on the blue side of the excitation wavelength 640 nm. This involves

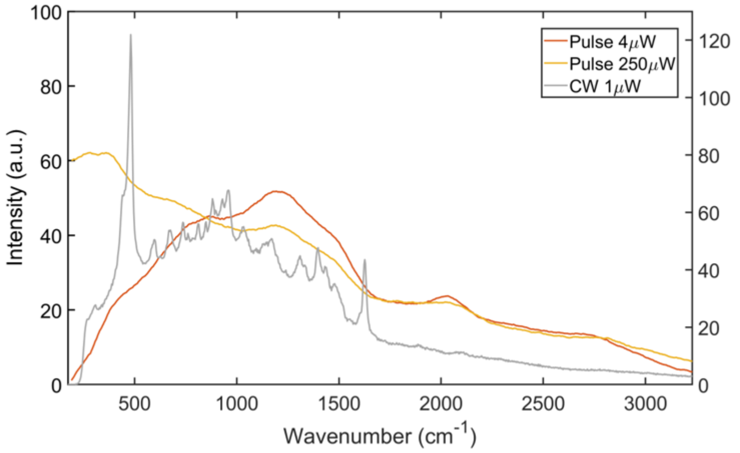

FIG. S4. Comparison of surface-enhance Raman scattering using a continuous wave (CW) excitation (wavelength = 633 nm) and emission from NPoM at different pulsed laser powers.

detecting the emitted light in the lower wavelength region beyond a short pass filter, that attenuates the spectrally-broad excitation laser ($>$ 10 nm bandwidth). The nonlinear emission also occurs, even with emission that has a higher energy than the excitation (Fig. S6. This suggests some form of heating, leading to emission from more-highly excited states, as discussed above. However, the thermal effect does not have a significant influence on reshaping the morphology of the nanoparticles. An evidence for this is a consistent darkfield scattering spectra from low to high power ranges (Fig. S7).

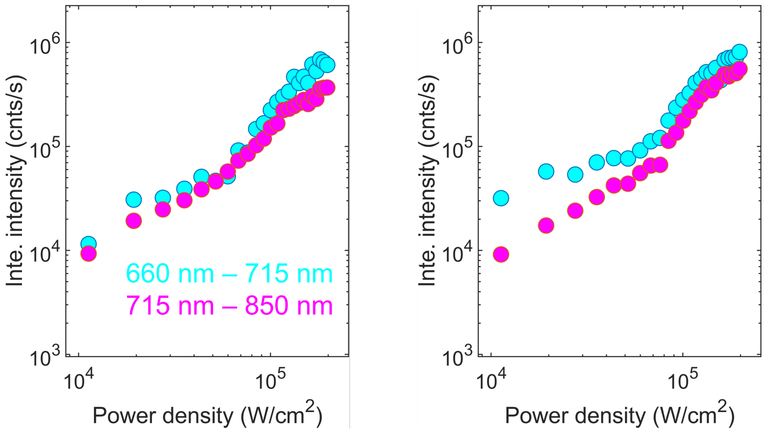

FIG. S5. Integrated intensity vs power density from two different NPoM cavities. The total emission intensities integrated across two spectral regions are shown for comparison.

C. Fitting results

As described in the Methods section, we fit the theoretical result to the experimental data to determine relevant parameters. We divide parameters into three classes:

A. Parameters which we fix globally over all NPOMs. In this class is Γ_z, which is assumed an intrinsic property of the molecules.

B. Parameters which are constant over NPOMs but allowed to vary according to the concentration of emitters (and thus target $\bar{N}$). In this class are $\Gamma_\downarrow, \kappa$.

C. Parameters which vary between each NPOM. In this class is the value g and an output intensity scaling which we denote S_{out}. This output scaling relates the measured output counts C_{out} to the theoretical photon number

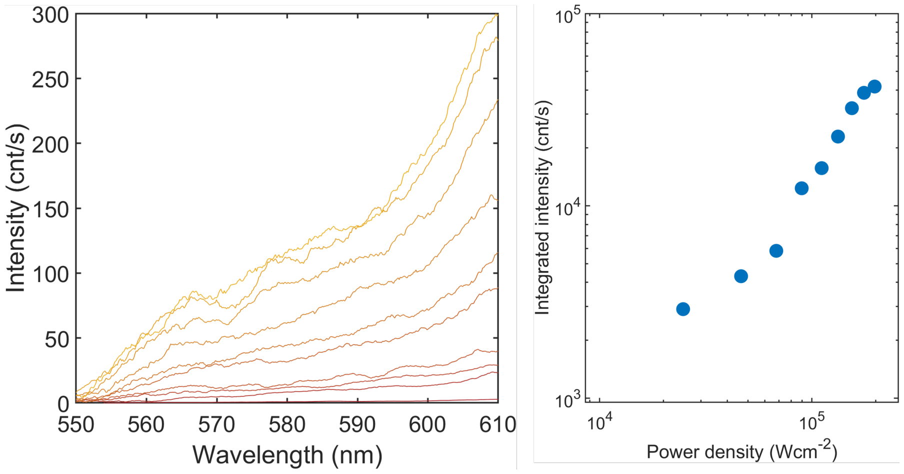

FIG. S6. The emission spectra on the blue side of the excitation wavelength at 640 nm (left). The integrated intensity over the spectra vs power density (right).

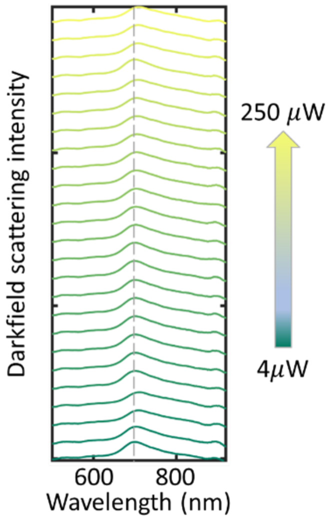

FIG. S7. NPoM cavity dark-field scattering spectrum measured after each emission measurement, for different laser powers.

n vias $C_{\text{out}} = S_{\text{out}} n$. In addition, we allow the value N to vary, within 20% of the target $\bar{N}$, to account for variation in the number of emitters actually coupled.

To perform the fitting, for each value of parameters in class B we optimise over the parameters in the class C (by simple least squares), and then calculate a reduced χ^2_R value from the match of the theory to the data when using those class-B parameters. We repeat this over a range of values of class-B parameters. This gives us a map of χ^2_R (see Fig. S8 for examples) from which we choose the optimal class-B parameters. We then use these parameters, along with the standard least squares fit of parameters in class C. Plotting the χ^2_R map allows us to identify how tightly confined are the class-B parameters. Following a Gaussian error model, 68 % of fits should have values of χ^2_R satisfying

$$\chi^2_R \leq \tilde{\chi}^2_R = \chi^2_{R,min} + \frac{1}{k_{\text{eff}}}\Delta \tag{S1}$$

and finding the area in parameter space defined by this inequality allows us to estimate errors. Here, k_{eff} and Δ are the reduced number of values and p-value from a χ^2 distribution table. This area is shown by grey contour in Fig. S8.

In performing this fitting, we take the theoretical parameter $\Gamma_\uparrow = g^2 \times (Powerdensity)$, where Power density is the measured value in units of Wcm^{-2}. The theoretical behaviour depends only on dimensionless ratios of the parameters $\Gamma_\uparrow, \Gamma_\downarrow, \Gamma_z, \kappa, g$, in order to produce the dimensionless photon number n. As such, the somewhat unusual units for $\Gamma_\uparrow$

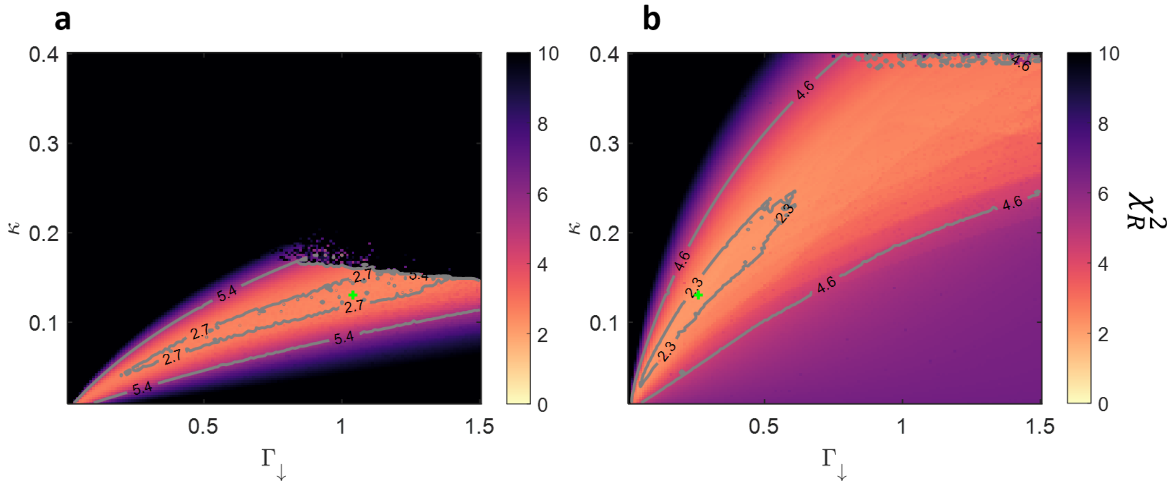

FIG. S8. Reduced χ^2 maps obtained by optimizing the values of $\Gamma_\downarrow, \kappa$ for a fixed value of $\Gamma_z = 10$ for N=5 (a) and N=10 (b).The green cross shows the minimum value of the χ_R^2 and the grey curves are the contour lines of the 1σ and 2σ confidence intervals. As discussed in the text, the units of these parameters are arbitrary, and only (dimensionless) ratios matter for the fitting.

do not cause a problem. Physical units could be restored by introducing an extra scaling parameter between power density and $\Gamma_\uparrow$, however since the theoretical fits depend only on ratios of parameters, the fit would be degenerate in this parameter.

A subset of the measured input-output curves appear to show different behaviour, with a saturation of the output power occurring below the lasing threshold. As such, these datasets cannot be fitted to the theoretical model. The origin of this different form of behaviour may be physical or instrumental, which needs further investigation. The fit to these datasets are considered invalid, since the extracted fitting parameters give erroneously high values. Thus, we consider only fits where $\chi_R^2 \leq \tilde{\chi}_R^2$. These bad fits account for $< 30\%$ of the entire dataset shown in Fig. S2 and the rest of the dataset shows the expected input-output curves of a laser.

There is no correlation between the fitting parameters in the class C [Figs. S9(a-c)], for $\chi_R^2 \leq \tilde{\chi}_R^2$. This lack of correlation is expected, since the coupling between emitters and cavity mode, and the out-coupling of the cavity mode to the detector should be independent parameters. There is however a correlation between the outcoupling intensity scaling and the emission below threshold [Figs. S9(d-f)], which implies that an efficient outcoupling efficiency of NPoM cavity results into a high emission intensity.

D. Shape of input-output curve

As mentioned in the main text, the shape of the input-output curve is not fixed when one goes beyond the weak-coupling laser theory of Ref. [2]. In the weak coupling limit, the parameter β fully determines the shape of the curve, while other parameters merely rescale the photon number or input power. In contrast, for strong coupling—particularly combined with inhomogeneous coupling strengths g_i—the shape can vary.

One way to characterise the variation of input-output curve shape is to consider the relationship between slope ratio β and the 'width' of the transition. As discussed in the main text, we define β by the ratio of slopes below and above threshold. We define transition width by considering the curvature of the input-output curve; the curvature should vanish in both the normal and lasing phases (where output is proportional to input). As such, we can use the peak curvature to numerically locate the critical power P_c, and the half-width half-maximum of curvature as a transition width ΔP. We use the right half-width to avoid problems when the transition width becomes comparable to the critical power. With these defined, we can calculate a dimensionless parameter $P_c/(\Delta P)$ which is a characteristic of the lasing transition. Figure S10 shows the relation between the dimensionless parameters β and $P_c/(\Delta P)$.

If the input-output curve were of fixed shape, there would exist a one-to-one relationship between β and $P_c/(\Delta P)$. We see first that the relationship predicted by our model without disorder differs from that of Ref. [2]. We note further (not shown) that this relationship is now parameter dependent, and would change depending on, e.g. $\Gamma_z/\Gamma_\downarrow, \kappa/\Gamma_\downarrow$. More importantly, the one-to-one relation clearly breaks down when we consider inhomogeneous couplings, with varying g_i. This demonstrates that the input-output curve does not in general have a fixed shape.

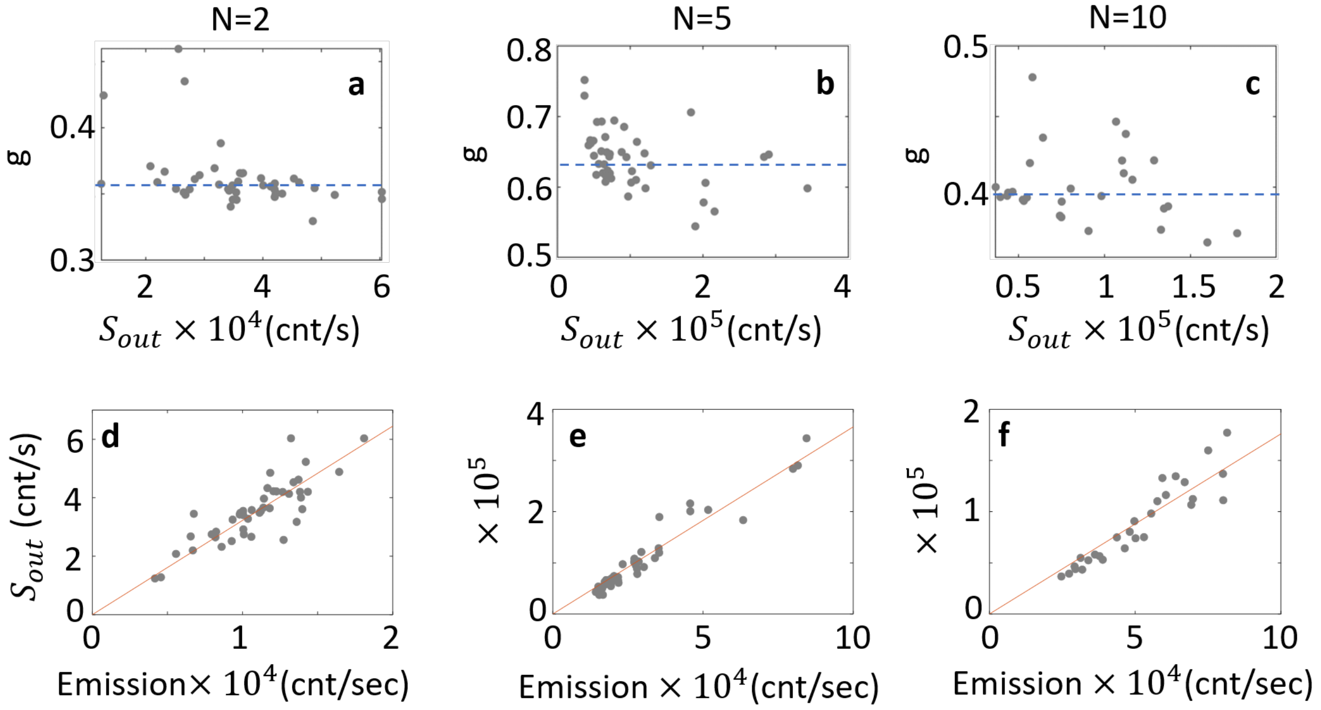

FIG. S9. (a-c) The extracted coupling strength g *vs* intensity scaling, showing uncorrelation between the parameters as indicated by the guide-to-the-eye blue line. (d-f) Intensity scaling *vs* emission below threshold and the orange curve is a linear fit.

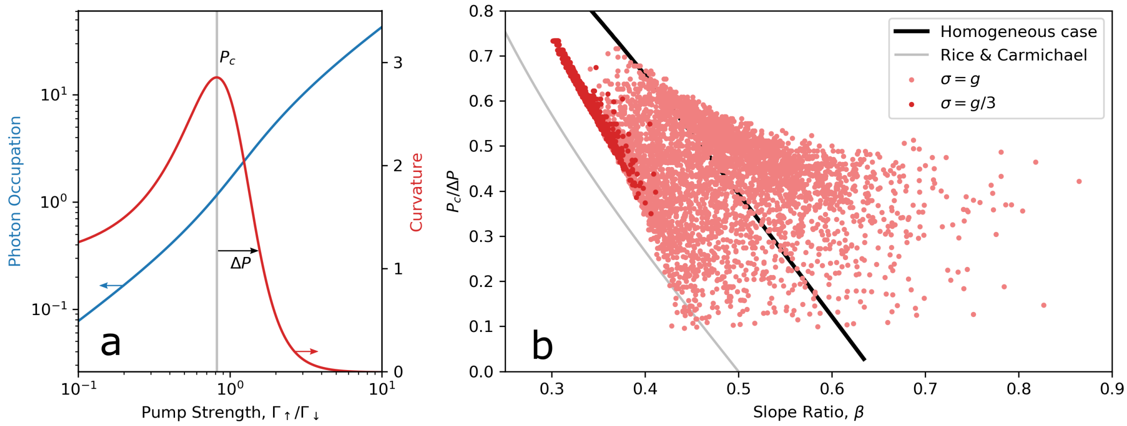

FIG. S10. Variation of shape of input-output curve. (a) Definition of transition width, extracted from the ratio of critical power P_c as defined by peak curvature, to right half-width half-maximum of curvature ΔP. Here curvature is the second derivative of input–output curve. (b) Relation between transition width, $P_c/(\Delta P)$, and slope-ratio β. The thick black line indicates the homogeneous limit of our model (no disorder in couplings. The gray line instead shows the weak-coupling formula of Ref. [2]. The points correspond to calculations with disordered couplings g_i, choosing g_i drawn from a distribution with standard deviations as indicated in the legend. Notably, with this disorder, there is no longer a one-to-one relation between transition width and slope ratio: i.e. the shape of the input–output curve varies. Parameters used (matching Fig. 2(i,j)): $\Gamma_z = \Gamma_\downarrow, \kappa = 1.74\Gamma_\downarrow$. In the homogeneous case, a range of values of g are considered to map out the range of β. In the inhomogeneous cases, we fix the root mean square of g_i to be $1.5\Gamma_\downarrow$, and sample distributions with widths as indicated in the legend.

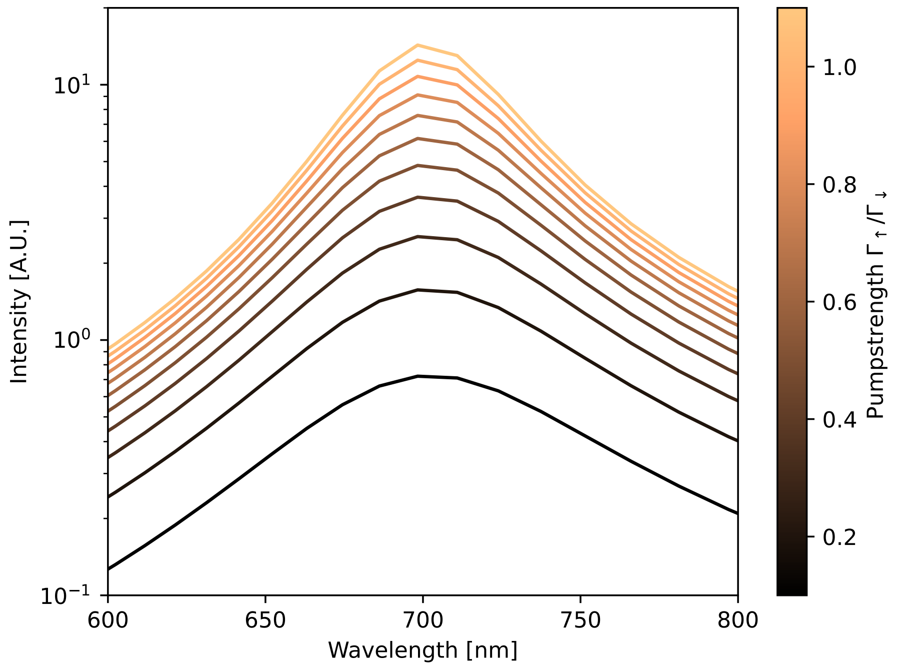

FIG. S11. The spectra calculated using exact steady state dynamics for different pump strengths (legend) along the lasing transition. As we can see, there is not substantial narrowing of the linewidth. The parameters used where as follows: N=4, $\kappa = 0.13, \Gamma_\downarrow = 1.1, \Gamma_z = 10, g = 0.62$. The photon subspace was truncated to 8 photons.

E. Linewidth calculations

To study how the linewidth of the spectra is expected to behave theoretically, we need to use more accurate numerical approaches, beyond the cumulant expansion we use for single-time expectations. Since we are specifically interested in the regime of few emitters we can use a numerically exact method based on permutation symmetry of N two-level systems coupled to a common mode. Such an approach has been used in various contexts, and can significantly reduce the computational workload required for exact calculations, see e.g. [28] for a review. We make use of the implementation available at Ref. [29]. Note that because of the permutation symmetry, we ignore disorder (e.g. varying coupling strengths) in this calculation.

When considering the lineshape, a key question (noted above) is how vibrational sidebands modify the lineshape and how this varies with pump strength. To capture this, we extend the code in Ref. [29] to consider m-level systems. To keep computational costs reasonable, we use $m = 4$, thus describing one vibrational excitation in addition to the two-level system.

Fig. S11 shows the results for parameters extracted from the fits in Fig. 2(e), but number of particles is kept at $N = 4$ and the photon subspace is truncated at 8 photons. The spectra show that even when we see a lasing-like threshold in the input-output curves, it does not lead to a substantial narrowing of the linewidth. This is due to the small number of photons present even after the lasing threshold.

1. *Effects of mode competition between plasmonic modes*

An important aspect of lasing behaviour is that lasing leads to an increase of stimulated emission into the lasing mode over other channels. In a system such as the one we study it is therefore important to look at the effects of competition with other plasmonic modes. To do this we consider here a model where we include three plasmonic modes. Thus, in addition to coupling to the $(1, 0)$ mode, we also add the degenerate $(1, 1)$ and $(1, -1)$ modes[17]. We then consider how the mode purity varies as we increase pumping through the lasing transition.

The Hamiltonian describing this system can be modelled with a generalisation of the Hamiltonian in Eq. (4), by

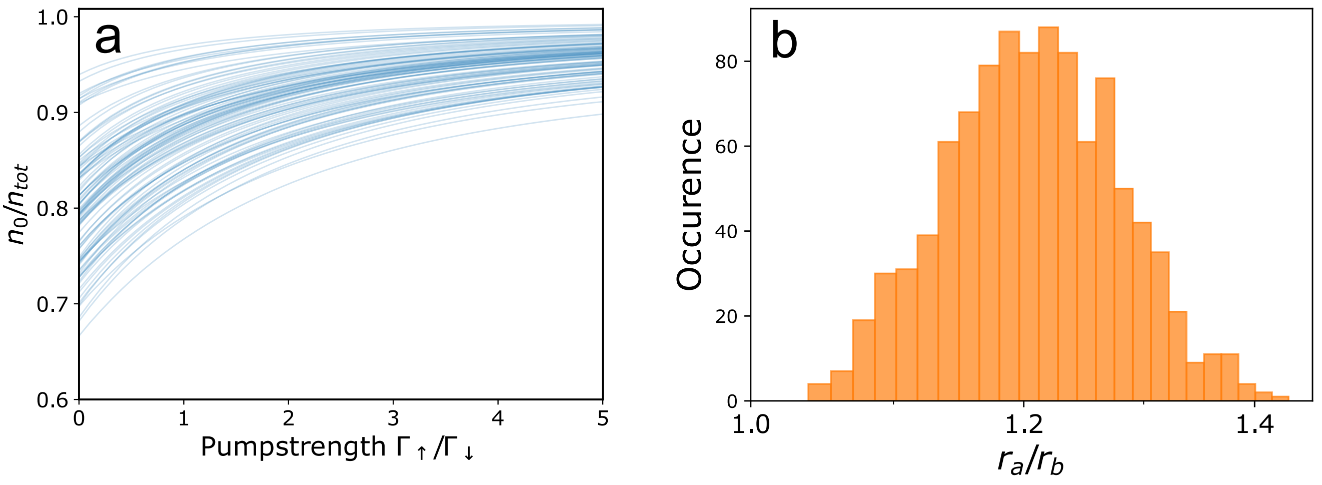

FIG. S12. (a) Mode purity of the lowest energy mode for 100 realisations of disorder in the coupling strength g. (b) Ratio of mode purity well above ($\Gamma_\uparrow = 5\Gamma_\downarrow$) and bellow ($\Gamma_\uparrow = 0.001\Gamma_\downarrow$) threshold for 1000 realisations. A logarithmic x-axis is used to match Figure 3(g) in the paper. The pump strengths used correspond to the far left and far right values in (a). Parameters: $N = 5, \Gamma_\downarrow = 1.1, \kappa = 0.13, \Gamma_z = 10$.

adding an index k to $\omega_k, a_k^{(\dagger)}$ and $g_{k,i}$ to denote the plasmonic mode involved. To avoid complex coupling constants, we choose to work in a basis of linear rather than circular modes, thus in place of the degenerate $(1, \pm 1)$ modes, we consider their symmetry (+) and antisymmetric (-) superposition (see Ref. [17])). As such, in the following we consider modes labelled by $k = 0, +, -$, with all coupling constants being real. This leads to equations analogous to before, but now $n_{k,k'}$ and $P_{k,i}$ now have k-dependencies.

With this extended model, we then solve the corresponding cumulant equations. Defining $\Delta_k = \omega_k - \epsilon, \gamma = \frac{1}{2}(\kappa + \Gamma_T), I_{k,i} = \text{Im}\left[P_{k,i}\right]$ we then find the solution takes the form:

$$P_{k,i} = \frac{1}{i\gamma - \Delta_k}\left[A_{k,i}\left(\sum_q g_{q,i} I_{q,i}\right)^2 + \sum_q B_{kq,i} I_{q,i} + C_{k,i}\right] \tag{S2}$$

$$A_{k,i} = \frac{8 g_{k,i}}{\Gamma_T(\Gamma_\uparrow + \Gamma_\downarrow)} \tag{S3}$$

$$B_{kq,i} = \frac{4 g_{q,i}}{\Gamma_\uparrow + \Gamma_\downarrow}\left[\frac{g_{k,i}}{2} + \sum_{k'}\left(\frac{i g_{k'i}}{i(\omega_k - \omega_{k'}) + \kappa}(\Pi^{(1)}_{k'k} - \Pi^{(1)*}_{kk'}) - \frac{g_{k'i}}{\Gamma_T}\text{Im}\left[\Pi^{(1)}_{kk'}\right]\right)\right] \tag{S4}$$

$$+ \frac{2 g_{k,i} g_{q,i} S_0}{\Gamma_T} - \frac{S_0 G_{kq}}{\Gamma_T} - \frac{4}{\Gamma_T(\Gamma_\uparrow + \Gamma_\downarrow)}\text{Im}\left[\Pi^{(3)}_{kq}\right] \tag{S5}$$

$$C_{k,i} = g_{k,i}\frac{S_0 + 1}{2} + S_0 \sum_{k'}\left(\frac{i g_{k'i}}{i(\omega_k - \omega_{k'}) + \kappa}(\Pi^{(1)}_{k'k} - \Pi^{(1)*}_{kk'}) - \frac{g_{k'i}}{\Gamma_T}\text{Im}\left[\Pi^{(1)}_{kk'}\right]\right) \tag{S6}$$

$$\Pi^{(1)}_{kk'} = \sum_i g_{k,i} P_{k'i} \tag{S7}$$

$$\Pi^{(3)}_{kk'} = \sum_{iq} g_{k,i} g_{k'i} g_{q,i} P_{q,i} \tag{S8}$$

$$S_0 = \frac{\Gamma_\uparrow - \Gamma_\downarrow}{\Gamma_\uparrow + \Gamma_\downarrow}. \tag{S9}$$

The values of the coupling constants $g_{i,k}$ are found by considering random locations for site i, and calculating

the mode profiles (following Ref. [17]. These equations are then solved numerically using an iterative approach. From such calculations we find the mode purity, and show the result of this in Fig. S12.

We define the mode purity as the ratio between occupation in the lowest energy mode to total occupation in all 3 modes. We find that the mode purity always increases when going through the threshold-like behaviour. Fig. S12(a) shows how the mode purity increases with pump for 1000 disorder realisations, while Fig. S12(b) shows the ratio of mode purity above and below threshold. Note that as the mode purity is already relatively high even bellow threshold, meaning that the radiation is already relatively well coupled to the lasing mode before collective effects happen.

F. Modelling bimolecular quenching

In the Letter, we saw that for the NPOM with the largest number of emitters, there was a reduction in output intensity at high pump powers, something not predicted by the model we consider. One possible explanation of such effects could be bimolecular quenching processes, where two excitations in close proximity interact in such a way that the excitations are lost. In this section we explore the consequence of adding such terms to our model.

We can account for bimolecular quenching processes in the model by including a non-linear incoherent term that causes two molecular excitations to dissipate: $\sum_{i\neq j} Q\mathcal{D}\left[\sigma_i^- \sigma_j^-\right]$ to Eq. (5). Within our cumulant equation approach, this leads to the addition of the following terms to the equations of motion

$$\partial_t S = \ldots - Q(N-1)\left(S+1\right)^2 \tag{S10}$$

$$\partial_t P = \ldots - \frac{Q}{2}(N-1)P(S+1) \tag{S11}$$

$$\partial_t D = \ldots - Q(N-2)D(S+1) \tag{S12}$$

These terms mean faster decay both of molecular excitation population and of coherences. In both cases, these extra decay rates increase both with the number of emitters N and with the inversion S.

The dependence on inversion seen in the equations above could potentially lead to suppression of lasing at high pump rates. However, the presence of additional decay will in general mean that the threshold-like behaviour will shift to higher pump strengths, and the shift will be larger for larger N. In Fig. S13 we show how different quenching strengths Q affect the input-output curves at $N = 5, 10$ and 25. We see that for strong enough quenching ($Q = 0.1$), the lasing threshold is significantly shfited for $N = 25$ while staying in place for the smaller N. We may note however that the shape of the $N = 25$ curve does not match those in Fig. 2g.

The mode of quenching we consider here assumes all-to-all quenching effect between different emitters. In a more realistic model one might assume that the distance between molecules would play a role, where reduced distance would increase the quenching effect so that higher densities would be expected to lead to even higher quenching compared to lower density realisations. However, in such a case, the results would vary significantly depending on the relative separation of emitters. Since this distance is not known for experiments it is hard to make a meaningful comparison with such a model.

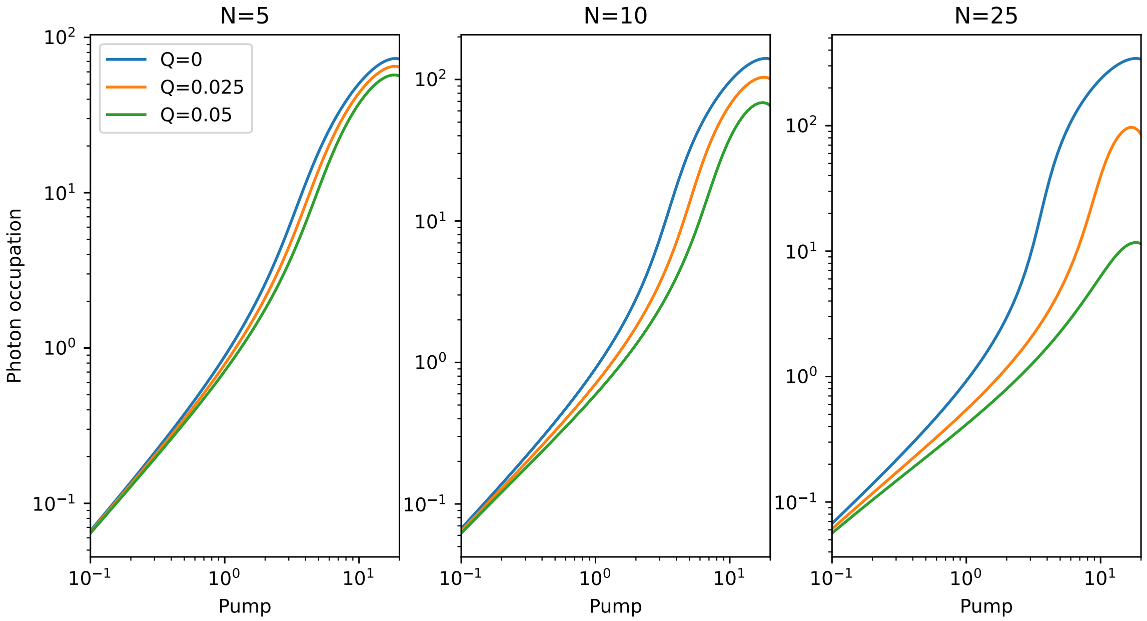

FIG. S13. Effect of bimolecular quenching on the input-output curves for various values of number of emitters N. All parameters are as in Figure 1, other than the extra quenching rate Q which takes the values as indicated in the legend.
